# Long working hours and increased risks of lean non-alcoholic fatty liver disease among Korean men and women

**DOI:** 10.1038/s41598-023-39154-x

**Published:** 2023-07-28

**Authors:** Ga-Young Lim, Yoosoo Chang, Inah Kim, Seungho Ryu, Ria Kwon, Jaechul Song

**Affiliations:** 1 Center for Cohort Studies, Total Healthcare Center, Kangbuk Samsung Hospital, Sungkyunkwan University School of Medicine, Seoul, 04514 Korea; 2 Institute of Medical Research, Sungkyunkwan University School of Medicine, Suwon, 16419 Korea; 3 Department of Occupational and Environmental Medicine, Kangbuk Samsung Hospital, Sungkyunkwan University School of Medicine, Samsung Main Building B2, 250, Taepyung-Ro 2Ga, Jung-Gu, Seoul, 04514 Korea; 4 Department of Clinical Research Design and Evaluation, Samsung Advanced Institute for Health Sciences and Technology, Sungkyunkwan University, Seoul, 06355 Korea; 5 Hanyang University Graduate School of Public Health, Seoul, 04763 Korea; 6 Department of Occupational and Environmental Medicine, Hanyang University College of Medicine, Seoul, 04763 Korea; 7 Department of Occupational and Environmental Medicine, College of Medicine and Graduate School of Public Health, Hanyang University, 222 Wangshimni-Ro, Seoul, 04763 Korea

**Keywords:** Diseases, Endocrinology, Health occupations, Risk factors

## Abstract

Despite the increasing prevalence of lean nonalcoholic fatty liver disease (NAFLD), its risk factors are not well established. We examined the association between long working hours and incident NAFLD in lean Korean workers with emphasis on sex-based effect modification. This cohort study involved 46,113 non-overweight (BMI < 23 kg/m^2^) and NAFLD-free Korean workers (mean age, 35.5 years). Working hours were categorized into 35–40 (reference), 41–52, and ≥ 53 h. The presence of fatty liver and its severity were determined using ultrasonography and NAFLD fibrosis score (NFS), respectively. Hazard ratios (HRs) and 95% confidence intervals (CIs) were estimated using parametric proportional hazards models. Incident cases of 5901 lean NAFLD developed over a median follow-up of 3.8 years. The incidence of lean NAFLD increased with increasing working hours with a stronger association in men than in women (*P* for interaction < 0.001). For men, multivariable-adjusted HRs (95% CIs) for lean NAFLD in time-dependent models comparing working hours of 41–52 and ≥ 53 h compared to the reference category were 1.17 (1.07–1.28) and 1.25 (1.12–1.39), respectively. The excess relative risk of developing lean NAFLD with intermediate/high NFS was observed in working hours of 41–52 and ≥ 53 h with a corresponding HR of 1.66 (1.13–2.43) and 1.54 (0.94–2.51), respectively. Conversely, no significant associations were found between working hours and incidence of lean NAFLD in women. In conclusion, long working hours were significantly associated with an increased incidence of lean NAFLD and its severe form in men but not in women.

## Introduction

The nonalcoholic fatty liver disease (NAFLD) is a highly prevalent disease worldwide, affecting approximately 24% of the global population with its incidence on the rise^1–3^. NAFLD is closely associated with obesity and other metabolic diseases including type 2 diabetes, insulin resistance, and metabolic syndrome^4,5^. However, NAFLD is not limited to obese individuals; and recent meta-analyses have reported an increasing prevalence of lean NAFLD^6–8^. Notably, the highest prevalence of lean NAFLD has been reported in middle-aged people, and working-age individuals, in Asian countries where long working hours are prevalent^7,9^. Therefore, identifying distinctive risk factors for NAFLD in the lean population may facilitate the development of effective preventive measures to avoid the burden of this disease and its associated consequences.

In Korea, the burden of chronic liver disease and its economic impact, including healthcare expenditure and productivity loss, is significant in the socioeconomically active population aged 40–59^10,11^. In 2020, Korea ranked third among OECD countries in terms of the annual total working hours, with a total of 1967 h, and ranked fourth in the number of workers working more than 40 h per week^9^. Recently, long working hours have been identified as an independent risk factor for NAFLD^12–14^. However, the studies have not investigated the association between long working hours and lean NALFD, nor the potential sex differences in this relationship, despite the higher prevalence of NAFLD and its advanced form in men than in women^15–18^. Differenced in NAFLD prevalence by sex may be due to various factors such as sex hormones, behavioral patterns in alcohol and drug use, and susceptibility to similar risk factors^16–18^. Liver fibrosis is the most important histologic factor of liver-related outcomes including liver cirrhosis, hepatocellular carcinoma (HCC), and liver-related mortality^19–23^, yet none of the studies have reported the association between long working hours and NAFLD with fibrosis.

This study aimed to investigate the relationship of long working hours with the development of lean NAFLD and worsening of liver fibrosis score separately in men and women while considering the changing status of working hours and other confounders during the follow-up period.

## Results

Table 1 shows the general characteristics of the 46,113 NAFLD-free and lean participants (48.7% men) with a low NFS score at baseline. The rate of long working hours was higher among men than women. In Supplementary Table 1, when we looked at the general characteristics by working hours, we found that long working hours groups both men and women were more likely to be younger and unmarried and less likely to have comorbidities, including hypertension and diabetes. They also had lower levels of physical activity and sleep durations, and higher levels of sitting time, depressive symptoms, and stress scores. The baseline characteristics are presented by study endpoints (Supplementary Tables 2, 3).

**Table 1:** Baseline characteristics by sex among 46,113 participants without nonalcoholic fatty liver disease.

Characteristics	Overall	Men	Women	*p*
Number	46,113	22,444	23,669	
Age (years)	35.5 (± 6.6)	37.0 (± 6.9)	34.1 (± 5.9)	< 0.01
Married (%)	72.1	73.7	70.6	< 0.01
Income (≥ 400*, %)	68.4	63.2	72.7	< 0.01
Education (≥ college, %)	91.5	92.4	90.6	< 0.01
Center (Seoul, %)	63.5	59.0	67.7	< 0.01
BMI (kg/m^2^)	20.6 (± 1.5)	21.3 (± 1.3)	20.1 (± 1.6)	< 0.01
Hypertension (%)	3.6	5.9	1.4	< 0.01
Diabetes mellitus (%)	2.0	1.8	2.1	0.013
Stress (score)	17.2 (± 6.5)	16.5 (± 6.2)	17.7 (± 6.8)	< 0.01
Depression (%)	10.8	7.1	14.3	< 0.01
Current smoker (%)	17.7	33.6	2.0	< 0.01
Alcohol drinking (≥ 10 g, %)	17.0	28.5	6.1	< 0.01
HEPA (%)	11.8	15.3	8.5	< 0.01
Sleep duration (< 8 h, %)	86.0	90.6	81.7	< 0.01
Sitting time (≥ 8 h, %)	72.9	71.6	74.1	< 0.01
Working hours (≥ 53 h/week, %)	23.7	29.7	18.1	< 0.01
Working hours (hours/week)	49.3 (± 8.8)	51.2 (± 8.9)	47.4 (± 8.4)	< 0.01

*1000 KRW; BMI, body mass index; HEPA, health-enhancing physical activity.

During 179,124.8 person-years of the follow-up, 5901 participants developed lean NAFLD, (incidence rate, 32.9/10^3^ person-years). The median follow-up period was 3.8 years (interquartile range, 2.0–5.7 years). Compared to the reference group (working 35–40 h), the group with working 41–52 h had a significantly higher risk of developing lean NAFLD but not for the group with working ≥ 53 h when baseline working hours were only included the model (Table 2). However, after introducing time-dependent variables of working hours and other covariates, the HRs (95% CI) for developing lean NAFLD were 1.15 (1.06–1.24) and 1.25 (1.14–1.37) in the groups with working 41–52 h, and ≥ 53 h, respectively, compared with the reference group. Notably, the association between working hours and the development of lean NAFLD differed by sex, with a stronger association in men than in women in the time-dependent model (*p* for interaction < 0.001). In men, those working 41–52 h and ≥ 53 h had a higher incidence of lean NAFLD compared to the reference group, with corresponding multivariable-adjusted HRs (95% CIs) of 1.22 (1.05–1.42) and 1.16 (0.99–1.38), respectively. In time-dependent models including updated status of working hour category and other confounders as time-varying covariates, the aHRs (95% CIs) for developing lean NAFLD were 1.17 (1.07–1.28) and 1.25 (1.12–1.39) in men with working 41–52 h and ≥ 53 h, respectively, whereas the associations were not statistically significant in women.

**Table 2:** Development of lean NAFLD according to the working hour category in men and women.

Categories of working hours	PY	Incident cases	Incidence rate (Cases per 1000 PY)	Age-adjusted HR (95% CI)	Multivariable-adjusted HR (95% CI)^a^	HR (95% CI)^b^ in a model using time-dependent variables
Total						
35–40 h	40868.5	999	24.4 (22.9–26.0)	1.00 (reference)	1.00 (reference)	1.00 (reference)
41–52 h	94706.6	3239	34.2 (33.0–35.4)	1.08 (1.01–1.16)	1.17 (1.04–1.32)	1.15 (1.06–1.24)
≥ 53 h	43549.9	1,63	38.2 (36.4–40.1)	1.08 (0.99–1.17)	1.12 (0.97–1.28)	1.25 (1.14–1.37)
*p* for trend				0.046	0.099	< 0.01
Men						
35–40 h	12250.9	604	49.3 (45.5–53.4)	1.00 (reference)	1.00 (reference)	1.00 (reference)
41–52 h	50317.0	2630	52.3 (50.3–54.3)	1.06 (0.97–1.16)	1.22 (1.05–1.42)	1.17 (1.07–1.28)
≥ 53 h	27526.2	1431	52.0 (49.4–54.7)	1.04 (0.95–1.15)	1.16 (0.99–1.38)	1.25 (1.12–1.39)
*p* for trend				0.222	0.127	< 0.01
Women						
35–40 h	28617.5	395	13.8 (12.5–15.2)	1.00 (reference)	1.00 (reference)	1.00 (reference)
41–52 h	44389.5	609	13.7 (12.7–14.8)	1.11 (0.98–1.27)	1.11 (0.90–1.37)	1.13 (0.97–1.32)
≥ 53 h	16023.6	232	14.5 (12.7–16.5)	1.16 (0.98–1.36)	1.04 (0.80–1.37)	1.10 (0.88–1.39)
*p* for trend				0.133	0.561	0.036

NAFLD, non-alcoholic fatty liver disease; PY, person-years; HR, hazard ratio; CI, confidence interval.

*p* < 0.001 for the overall interaction between sex and working hour category for incident NAFLD (time-dependent model).

^a^Estimated from parametric proportional hazard models with age, year of visit, center, sex, marital status, income level, education level, stress level, hypertension, diabetes mellitus, depression, smoking status, alcohol consumption, physical activity level, sitting time, and sleep duration.

^b^Estimated from parametric proportional hazard models with center, sex, marital status, and education level as time-fixed variables and working hours, stress level, hypertension, diabetes mellitus, depression, smoking status, alcohol consumption, physical activity level, sitting time, and sleep duration as a time-dependent variable.

During the follow-up of 189784.4 person-years, 305 participants developed lean NAFLD with an intermediate/high NFS score, resulting in an incidence rate of 1.6/10^3^ person-years (Table 3). The median follow-up period was 4.0 years (interquartile range, 2.1–5.9 years). In models based on baseline levels of working hours, there were a tendency of increasing incidence of lean NAFLD with an intermediate/high NFS score, although this association was not statistically significant. In the time-dependent models, where changes in working hours and other confounders during the follow-up period were treated as time-varying covariates, the HRs (95% CIs) of developing lean NAFLD with an intermediate/high NFS score was 1.49 (1.06–2.10) for those with working 41–52 h and 1.39 (0.88–2.19) for those with working ≥ 53 h, compared with the reference group. This association was observed primarily in men as reliable estimates were not obtained in women due to the limited number of incident cases. The association between working hours and the incidence of lean NAFLD tended to differ by sex in the time-dependent model, although not significant (*p* for interaction 0.058).

**Table 3:** Development of NAFLD plus intermediate/high probability of advanced fibrosis based on NFS according to the working hour category in men and women.

Categories of working hours	PY	Incident cases	Incidence rate (Cases per 1000 PY)	Age-adjusted HR (95% CI)	Multivariable-adjusted HR (95% CI)^a^	HR (95% CI)^b^ in a model using time-dependent variables
Total						
35–40 h	42444.3	65	1.53 (1.20–1.95)	1.00 (reference)	1.00 (reference)	1.00 (reference)
41–52 h	100611.6	153	1.52 (1.30–1.78)	1.16 (0.86–1.58)	1.49 (0.81–2.74)	1.49 (1.06–2.10)
≥ 53 h	46728	87	1.86 (1.51–2.30)	1.30 (0.93–1.81)	1.43 (0.69–2.96)	1.39 (0.88–2.19)
*p* for trend				0.185	0.869	0.116
Men						
35–40 h	13239.2	47	3.55 (2.67–4.72)	1.00 (reference)	1.00 (reference)	1.00 (reference)
41–52 h	55254.3	137	2.48 (2.10–2.93)	1.23 (0.87–1.74)	1.79 (0.87–3.64)	1.66 (1.13–2.43)
≥ 53 h	30311.4	78	2.57 (2.06–3.21)	1.33 (0.91–1.94)	1.96 (0.87–4.42)	1.54 (0.94–2.51)
*p* for trend				0.303	0.598	0.067
Women						
35–40 h	29205.1	18	0.62 (0.39–0.98)	1.00 (reference)	1.00 (reference)	1.00 (reference)
41–52 h	45357.3	16	0.35 (0.22–0.58)	0.87 (0.44–1.73)	0.87 (0.22–3.48)	–
≥ 53 h	16417.1	9	0.55 (0.29–1.05)	1.05 (0.47–2.35)	0.365 (0.007–17.458)	–
*p* for trend				0.811	0.460	–

NAFLD, non-alcoholic fatty liver disease; NFS, nonalcoholic fatty liver disease fibrosis score; PY, person-years; HR, hazard ratio; CI, confidence interval.

*p* = 0.058 for the overall interaction between sex and working hour category for incident NAFLD plus fibrosis based on NFS (time-dependent model).

^a^Estimated from parametric proportional hazard models with age, year of visit, center, sex, marital status, income level, education level, stress level, hypertension, depression, smoking status, alcohol consumption, physical activity level, sitting time, and sleep duration.

^b^Estimated from parametric proportional hazard models with center, sex, marital status, and education level as time-fixed variables and working hours, stress level, hypertension, depression, smoking status, alcohol consumption, physical activity level, sitting time, and sleep duration as a time-dependent variable.

## Discussion

This study demonstrated an association between long working hours and the risk of developing lean NAFLD and NAFLD plus intermediate/high liver fibrosis score among 46,113 lean Korean workers, separately from men and women. Long working hours were associated with an increased risk of developing lean NAFLD and NAFLD plus fibrosis, especially when the updated status of working hours and other factors during the follow-up were treated as time-varying covariates, but these patterns were observed only in men but not women.

At baseline, both men and women with long working hours showed lower comorbidities, such as diabetes mellitus and hypertension, but had higher prevalence of unhealthy lifestyles compared to those with lower working hours. This pattern is consistent with finding from a study conducted by Artazcoz et al.^24^, which reported that long working hours were associated with short sleep duration, poor mental health, smoking, and a lack of physical activity, all of which contribute to unhealthy lifestyles. However, the specific risk factors associated with unhealthy lifestyles for lean NALFD, particularly among Asian individuals of working age, remain poorly understood.

While several studies have investigated long working hours as a risk factor for NAFLD in the general population^12–14^, few have examined this relationship in the lean population. Li et al.^25^ reported that the lean NAFLD group had a higher prevalence of overtime work (> 40 h/week) but a shorter sleep duration than the control group. However, previous studies were limited by either unclear temporal relationship due to the cross-sectional study design or a lack of separate analyses of men and women. Given that sex differences can affect lifestyle habits and the onset and course of chronic diseases, including NAFLD^16,26,27^, as well as physiological functions and disease susceptibility^16,28^, it is crucial to investigate this relationship by sex. Our study found that the association between long working hours and lean NAFLD was stronger in men than in women, and the risk increased progressively with longer working hours. Moreover, among men, those who worked 41–52 h or ≥ 53 h had a higher risk of incident lean NAFLD with fibrosis than those who worked 35–40 h, while no significant association was observed in women. This result was similarly reported in a study conducted by Song et al., who reported an association between long working hours and abnormal liver function test only in men^29^. Women in our study had a lower mean age and had better health conditions such as lower BMI and lower prevalence of hypertension, as well as better health behaviors, including lower smoking, drinking, and longer sleep time, than men. These factors may have contributed to the lack of significant association between long working hours and lean NAFLD incidence in women. Furthermore, previous research has shown that postmenopausal women experience hormonal changes that can affect disease occurrence differently than premenopausal women^16,27,30^. Premenopausal women may benefit from the biological protective effect of estrogen, which reduces fat peroxidation and may exhibit anti-inflammation effect^31,32^. However, our study did not consider postmenopausal women, so future research should address this issue to confirm our findings.

Furthermore, it is also important to consider the potential influence of women's specific job characteristics on the observed relationship, as well as the additional responsibilities they often shoulder for household chores and childcare. According to the Statistics Korea's Annual Report on the Economically Active Population Survey for 2018^33^, the sector with the highest percentage of women employed, surpassing men, is health care and social services sector (81.5%), followed by accommodation and food services (58.7%). Within the health and social services sector, which had the highest percentage of women, women tended to have shorter working hours (157.2 h per month, including actual hours worked and overtime) compared to men (166.3 h per month) in the same year. Similarly, in the accommodation and food services sector, which ranked second among female employment, women worked fewer hours per month (172.2 h per month) compared to men (177.8 h per month)^33^. These findings indicate that even in industries with a high representation of women, women tend to work shorter hours than their male counterparts, suggesting a relatively lower exposure to the risk of long working hours.

Conversely, it is also crucial to acknowledge that the majority of women bear the responsibility of household chores and childcare after work^34^ despite the lack of available statistics specifically for the economically active population. However, due to limited data availability in our study, we were unable to incorporate information regarding women's occupational characteristics or household chores, potentially impacting their overall workload. This highlights the necessity for future research to encompass such information to further validate our findings.

Recent studies have identified genetic and clinical risk factors for lean NAFLD including the association of the PNPLA3 rs738409 gene^35–38^, impaired glucose tolerance, and unfavorable adipokine profiles characterized by low adiponectin concentrations^37^. Increased physical inactivity^38^ and decreased leisure time physical activity, which are increased with long working hours, are also associated with lean NAFLD^39,40^. In addition, prolonged working long hours have been shown to cause job-related psychosocial stress which can lead to an inflammatory response in the liver as reported by previous studies^41–44^. Moreover, long working hours are also associated with poor dietary behaviors, including high consumption of simple sugars, skipping breakfast, eating out, consuming instant food, overeating, and fast eating^45^. However, since our study did not include the information on dietary behaviors, there is a possibility of unmeasured confounding in the observed associations. Future research is needed to clarify the mechanism of lean NAFLD development associated with long working hours while considering dietary behaviors as well as including quantitative and qualitative assessment of diet.

Our study had several limitations. First, we used abdominal ultrasonography to diagnose fatty liver instead of liver biopsy. Although ultrasonography is a widely accessible and noninvasive imaging technique for detecting fatty liver and has been proven to show a reliable and accurate detection of moderate-to-severe hepatic steatosis compared with liver biopsy^46^, it may still have limitations in detecting mild hepatic steatosis. Second, we used noninvasive fibrosis indices (NFS) to define liver fibrosis in patients with NAFLD. Although this score has limited performance than liver biopsy in predicting changes in fibrosis, it has consistently demonstrated its ability to predict liver-related morbidity and mortality in previous studies^47,48^. Third, our dataset was limited in the number of visits available, with an average number of visits 2.6 (± 1.72) and approximately 60% of participants had fewer than 3 visits, limiting analyses that considered changes in working hours on the risk of lean NAFLD. We presented the results in time-dependent analyses, where updated information on working hours and covariates were treated as time-varying covariates. However, it is important to examine the impact of changes in working hours on incident NAFLD. Further research is warranted to specifically investigate the impact of variations in working hours on NAFLD. Fourth, we could not consider the information on job characteristics or job position, which may influence the effect of working hours on the risk of lean NAFLD. Furthermore, our study only included day workers than shift workers, so there is a possibility of residual and unmeasured confounding in observed association between long working hours and risk of lean NAFLD. Finally, our study was limited to young and middle-aged Koreans, so our results have limitations in generalizing to other population groups.

In this large-scale cohort study of relatively young, working population with repeated measurements of working hours, liver ultrasound and wide range of covariates, we found a longitudinal and independent association between long working hours and the risk of lean NAFLD (both overall NAFLD and more advanced form based on liver fibrosis score) in men but not in women. Future studies are needed to understand the differential effect of working hours on the lean NAFLD risk by sex and to confirm that preventive measures to avoid excessive working hours may help reduce the incidence of NAFLD and its consequences even in a young, healthy and non-overweight working population.

## Methods

### Study population

The Kangbuk Samsung Health Study is a cohort study that involved Korean adults aged 18 years or older who received annual or biennial health screenings at the Kangbuk Samsung Hospital Health Screening Centers in Seoul or Suwon, Republic of Korea^49^. For this study, we restricted 135,272 participants who underwent comprehensive examinations from 2011 to 2017 and participated in at least one follow-up examination by the end of 2018 and who were not overweight and were not shift-workers, and had information on their working hours. Exclusion criteria included missing data on body mass index (BMI); overweight defined as BMI of ≥ 23 kg/m^2^; fatty liver on ultrasound at baseline; history of liver cirrhosis or liver cirrhosis on ultrasound; history of liver disease or use of medications for liver disease; positive serologic markers for hepatitis B or C virus; history of cancer; history of heart disease, alcohol intake of ≥ 30 g/day for men and ≥ 20 g/day for women; intermediate or high fibrosis score based on NAFLD fibrosis score (NFS) at baseline; or use of steatogenic medication such as amiodarone, tamoxifen, methotrexate, valproate, or corticosteroids within the past month. This study was approved by the Institutional Review Board of Kangbuk Samsung Hospital (IRB No. 2021-01-034), which waived informed consent as we used only de-identified data routinely collected as part of a routine health screening program. All procedures involved in this study of human participants were in accordance with the ethical standards of the institutional research committee and with the 1964 Helsinki declaration and its later amendments or comparable ethical standards.

### Measurements

Abdominal ultrasonography, blood tests, and physical examinations were performed as a basic part of the health check-up program at baseline and follow-up visits. Data on demographic factors, socioeconomic status, health behaviors, sleep duration, medical history, and medication use were collected using standardized, structured, self-administered questionnaires^49^. The participants’ physical activity and sitting time were evaluated using the validated Korean version of the International Physical Activity Questionnaire-Short Form, and classified as inactive, minimally active, or health-enhancing physical active (HEPA). HEPA was defined as either: (1) vigorous-intensity activity on ≥ 3 days per week accumulating ≥ 1500 metabolic equivalent task min/week or (2) 7 days of any combination of walking, moderate-intensity, or vigorous-intensity activities achieving at least 3000 MET min/week^50^. Depressive symptoms were assessed using the Korean version of the Center for Epidemiologic Studies Depression scale (CES-D) and categorized as CES-D scores < 16 (no depressive symptoms) and ≥ 16 (the presence of depressive symptoms), which has been validated in previous studies^51,52^.

BMI was calculated as body weight divided by height squared (kg/m^2^). We applied the BMI cut-offs of ≥ 23 kg/m^2^ to define overweight for adult Asians; accordingly, being non-overweight or lean was defined as a BMI of < 23 kg/m^2^ ^53^.

Hypertension was defined as BP ≥ 140/90 mmHg or current use of BP-lowering agents^54^. Type 2 diabetes mellitus was defined as fasting serum glucose ≥ 126 mg/dL, hemoglobin A1c ≥ 6.5%^55^, or current use of insulin or glucose-lowering medications.

### Definition of long working hours

Long working hours were assessed using a self-administered questionnaire in Korean, both at baseline and follow-up visits. Working hours were defined as a continuous variable based on responses to a single question such as “During the last year, what were your average working hours per week.” The working hours were then categorized into three groups: (1) 35–40 h (reference group), (2) 41–52 h, and (3) ≥ 53 h. In Korea, the standard working hours are 40 h per week, with extensions up to 52 h per week permitted with the worker’s consent, as per the Labor Standard Act^56^. Therefore, the reference group was defined as those working within the standard working hours (35–40 h). The risk groups were classified into two categories: the 41–52 h group and the 52 h or more group, which exceeded the legal working hours.

### Study endpoint: lean NAFLD and lean NAFLD with intermediate/high fibrosis score

**Figure 1:**
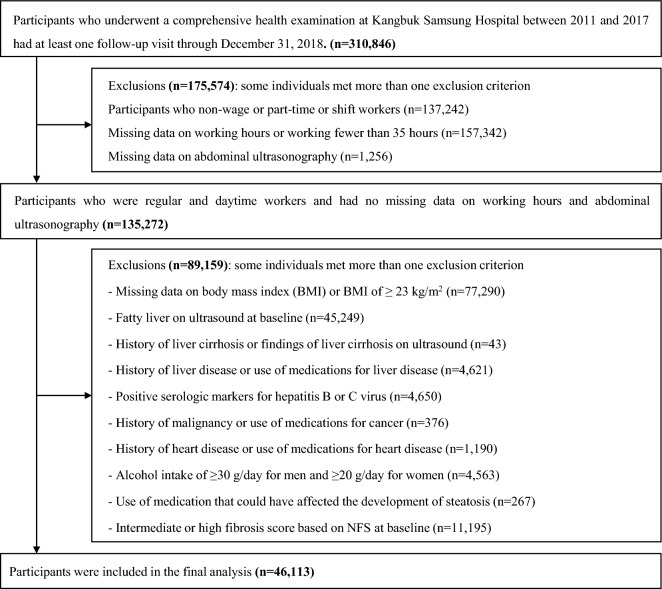
Flowchart of study participants.

The determination of fatty liver was conducted through abdominal ultrasonography, carried out by experienced radiologists who were blinded to the study's objective. Standard criteria were used, including the presence of a diffuse increase in fine echoes in the liver parenchyma compared with kidney or spleen parenchyma, deep beam attenuation, and bright vessel walls^57^. The diagnosis of fatty liver had substantial inter-observer reliability (kappa statistic of 0.74) and excellent intra-observer reliabilities (kappa statistic of 0.94)^49^. We defined NAFLD as the presence of fatty liver on ultrasonography in the absence of excessive alcohol consumption (a threshold < 20 g/day for women and < 30 g/day for men) and other identifiable causes of hepatic steatosis (see further details in the exclusion criteria in Fig. 1)^58^.

The Nonalcoholic fatty liver score (NFS), a useful non-invasive tool for identifying a high probability of advanced liver fibrosis in NAFLD patients,^59^ was used to assess the risk of severe NAFLD. The NFS was calculated using the following formula: NFS = − 1.675 + 0.037 × age (year) + 0.094 × BMI (kg/m^2^) + 1.13 × impaired fasting glycemia or diabetes (yes = 1, no = 0) + 0.99 × AST/ALT ratio − 0.013 × PLT (× 10^9^/L)  − 0.66 × albumin (g/dL). Participants were categorized into low (NFS < − 1.455), intermediate (NFS: 0.676 to − 1.455), and high (NFS > 0.676) risk groups according to their probability of advanced liver fibrosis^47^.

Lean NAFLD was defined as the presence of NAFLD in individuals with a BMI of < 23 kg/m^2^. Furthermore, lean NAFLD with intermediate/high NFS was defined as the presence of lean NAFLD accompanied by intermediate NFS or higher scores.

### Statistical analysis

The chi-square test and one-way analysis of variance were conducted to compare the characteristics of the study participants according to working hour category (35–40 h, 41–52 h, and ≥ 53 h). As there was a significant interaction by sex in the relationship between long working hours and the risk of lean NAFLD, we presented all the data separately for men and women. The outcomes were the development of lean NAFLD and lean NAFLD with intermediate/high liver fibrosis, as assessed using the NFS score. The incidence rate was expressed as the number of new-onset lean NAFLD cases per 1000 person-years. A parametric proportional hazards model was used to estimate hazard ratios (HRs) and 95% CIs for incident lean NAFLD. The models were initially adjusted for age and sex, and subsequently adjusted for year of screening examination, center (Seoul, or Suwon), marital status (unmarried, married, or missing), monthly household income (< 4, ≥ 4 million Korean won, or missing), education level (less than college graduate, college graduate or more, or missing), stress (yes, no, or missing), history of hypertension (yes, no, or missing), diabetes mellitus (yes, no, or missing), depressive symptoms (yes, no, or missing), smoking status (non-current smoker, current smoker, or missing), alcohol consumption (≤ 10, or > 10 g/day), physical activity (inactive, minimally active, HEPA, or missing), sitting time (< 8, ≥ 8 h or missing), and sleep duration (< 8, ≥ 8 h or missing). For covariates, missing values were treated as a separate category and were included in the statistical models. However, to address the issue of missing data, we also conducted reanalyses using a dataset that excluded observations with missing values.

To account for changes in working hours and confounding variables over time, we performed time-dependent analyses while introducing working hours and other covariates as time-varying covariates in the models. We assessed the proportional hazards assumption by examining graphs of estimated log (− log) survival and found no violation of the assumption. The likelihood ratio was used to evaluate interactions between sex (men vs. women) by comparing models with and without multiplicative interaction terms. We used STATA version 17.0 (STATA Corp LP, College Station, TX, USA) for all statistical analyses. All p-values were reported as two-tailed, and values less than 0.05 were considered statistically significant.

## Supplementary Information

Supplementary Tables.

## Data Availability

The data are not publicly available outside of the hospital because of Institutional Review Board restrictions (the data were not collected in a way that could be distributed widely). However, the analytical methods are available from the corresponding author upon request.
