# Supplementary material for: Long working hours and increased risks of lean non-alcoholic fatty liver disease among Korean men and women
Source: Sci Rep. 2023 Jul 28;13:12230. doi: 10.1038/s41598-023-39154-x (PMC10382542; doi:10.1038/s41598-023-39154-x)
Supplement: Supplementary file 1 — Supplementary Tables. [file 41598_2023_39154_MOESM1_ESM.docx]

Supplementary table 1. Baseline characteristics by working hours per week among 46,113 participants without nonalcoholic fatty liver disease.

| Characteristics | Overall | Working hours per week | | | | | | | |
| --- | --- | --- | --- | --- | --- | --- | --- | --- | --- |
|  |  | Men | | | p for trend | Women | | | p for trend |
|  |  | 35–40 hours | 41–52 hours | ≥53 hours |  | 35–40 hours | 41–52 hours | ≥53 hours |  |
| Number | 46,113 | 3,244 | 12,559 | 6,661 |  | 7,639 | 11,743 | 4,287 |  |
| Age (years) | 35.5 (±6.6) | 40.2 (±8.5) | 36.6 (±6.6) | 36.3 (±6.3) | <0.01 | 35.2 (±6.4) | 33.5 (±5.5) | 33.6 (±5.9) | <0.01 |
| Married (%) | 72.1 | 80.9 | 72.5 | 72.4 | <0.01 | 83.2 | 66.5 | 59.4 | <0.01 |
| Income (≥400*, %) | 68.4 | 64.9 | 62.1 | 64.6 | 0.553 | 75.4 | 71.3 | 71.4 | <0.01 |
| Education (≥college, %) | 91.5 | 86.9 | 93.1 | 93.8 | <0.01 | 86.7 | 92.5 | 92.3 | <0.01 |
| Center (Seoul, %) | 63.5 | 64.2 | 58.0 | 58.5 | <0.01 | 62.7 | 68.2 | 75.1 | <0.01 |
| BMI (kg/m^2^) | 20.6 (±1.5) | 21.3 (±1.3) | 21.3 (±1.3) | 21.3 (±1.3) | 0.5543 | 20.1 (±1.5) | 20.0 (±1.5) | 19.9 (±1.5) | <0.01 |
| Hypertension (%) | 3.6 | 8.6 | 5.5 | 5.4 | <0.01 | 1.8 | 1.1 | 1.4 | 0.011 |
| Diabetes mellitus (%) | 2.0 | 1.9 | 1.9 | 1.6 | 0.212 | 2.3 | 2.1 | 1.8 | 0.079 |
| Stress (score) | 17.2 (±6.5) | 14.8 (±5.5) | 16.1 (±5.7) | 18.3 (±6.8) | <0.01 | 16.3 (±6.2) | 17.9 (±6.7) | 19.8 (±7.5) | <0.01 |
| Depression (%) | 10.8 | 5.7 | 6.1 | 9.6 | <0.01 | 10.9 | 14.2 | 20.8 | <0.01 |
| Current smoker (%) | 17.7 | 36.3 | 31.9 | 35.7 | 0.262 | 1.6 | 1.8 | 2.9 | <0.01 |
| Alcohol drinking (≥10 g, %) | 17.0 | 32.7 | 27.2 | 28.9 | 0.012 | 5.3 | 5.8 | 8.3 | <0.01 |
| HEPA (%) | 11.8 | 18.6 | 15.1 | 13.9 | <0.01 | 9.3 | 8.1 | 7.9 | 0.002 |
| Sleep duration (<8 hours, %) | 86.0 | 86.0 | 90.3 | 93.4 | <0.01 | 75.4 | 83.2 | 89.0 | <0.01 |
| Sitting time (≥8 hours, %) | 72.9 | 54.0 | 72.2 | 78.7 | <0.01 | 61.7 | 78.6 | 82.8 | <0.01 |

***** 1,000 KRW; Abbreviations: BMI, body mass index; HEPA, health-enhancing physical activity

Supplementary table 2. Baseline characteristics according to the development of NAFLD.

| Characteristics | Overall | Non-NAFLD | NAFLD | p |
| --- | --- | --- | --- | --- |
| Number | 46,113 | 40,212 | 5,901 |  |
| Age (years) | 35.5 (±6.6) | 35.2 (±6.6) | 37.4 (±6.4) | <0.01 |
| Married (%) | 72.1 | 71.4 | 76.5 | <0.01 |
| Income (≥400*, %) | 68.4 | 68.6 | 66.2 | 0.005 |
| Education (≥college, %) | 91.5 | 91.4 | 91.7 | 0.363 |
| Center (Seoul, %) | 63.5 | 63.5 | 62.9 | 0.429 |
| BMI (kg/m^2^) | 20.6 (±1.5) | 20.5 (±1.6) | 21.5 (±1.1) | <0.01 |
| Hypertension (%) | 3.6 | 3.2 | 5.9 | <0.01 |
| Diabetes mellitus (%) | 2.0 | 1.8 | 3.2 | 0.013 |
| Stress (score) | 17.2 (±6.5) | 17.1 (±6.5) | 17.1 (6.4) | 0.621 |
| Depression (%) | 10.8 | 11.1 | 8.5 | <0.01 |
| Current smoker (%) | 17.7 | 15.7 | 31.3 | <0.01 |
| Alcohol drinking (≥10 g, %) | 17.0 | 16.0 | 24.1 | <0.01 |
| HEPA (%) | 11.8 | 11.6 | 13.0 | 0.002 |
| Sleep duration (<8 hours, %) | 86.0 | 85.6 | 89.0 | <0.01 |
| Sitting time (≥8 hours, %) | 72.9 | 73.1 | 71.7 | 0.044 |
| Working hours (≥53 hours/week, %) | 23.7 | 23.1 | 28.2 | <0.01 |
| Working hours (hours/week) | 49.3 (±8.8) | 49.1 (±8.8) | 50.7 (±8.9) | <0.01 |

***** 1,000 KRW; Abbreviations: NAFLD, Non-alcoholic fatty liver disease; BMI, body mass index; HEPA, health-enhancing physical activity

Supplementary table 3. Baseline characteristics according to the development of NAFLD with fibrosis status.

| Characteristics | Overall | NAFLD with intermediate/high NFS | | p |
| --- | --- | --- | --- | --- |
|  |  | No | Yes |  |
| Number | 46,113 | 45,808 | 305 |  |
| Age (years) | 35.5 (±6.6) | 35.5 (±6.6) | 44.2 (±6.7) | <0.01 |
| Married (%) | 72.1 | 71.9 | 94.9 | <0.01 |
| Income (≥400*, %) | 68.4 | 68.3 | 82.7 | 0.002 |
| Education (≥college, %) | 91.5 | 91.5 | 86.3 | 0.002 |
| Center (Seoul, %) | 63.5 | 63.4 | 70.2 | 0.015 |
| BMI (kg/m^2^) | 20.6 (±1.5) | 20.6 (±1.5) | 21.8 (±1.0) | <0.01 |
| Hypertension (%) | 3.6 | 3.5 | 13.2 | <0.01 |
| Diabetes mellitus (%) | 2.0 | 2.0 | 2.3 | 0.671 |
| Stress (score) | 17.2 (±6.5) | 17.2 (±6.5) | 16.4 (±6.2) | 0.043 |
| Depression (%) | 10.8 | 10.8 | 9.1 | 0.342 |
| Current smoker (%) | 17.7 | 17.5 | 40.7 | <0.01 |
| Alcohol drinking (≥10 g, %) | 17.0 | 16.8 | 41.0 | <0.01 |
| HEPA (%) | 11.8 | 11.8 | 15.4 | 0.052 |
| Sleep duration (<8 hours, %) | 86.0 | 86.0 | 89.7 | 0.079 |
| Sitting time (≥8 hours, %) | 72.9 | 73.0 | 58.3 | <0.01 |
| Working hours (≥53 hours/week, %) | 23.7 | 23.7 | 28.5 | 0.136 |
| Working hours (hours/week) | 49.3 (±8.8) | 49.3 (±8.8) | 50.3 (±9.5) | 0.058 |

***** 1,000 KRW; Abbreviations: BMI, body mass index; HEPA, health-enhancing physical activity
